# Carbonation of Alkali-Activated Materials: A Review

**DOI:** 10.3390/ma16083086

**Published:** 2023-04-13

**Authors:** Ghandy Lamaa, António P. C. Duarte, Rui Vasco Silva, Jorge de Brito

**Affiliations:** CERIS (Civil Engineering Research and Innovation for Sustainability), Civil Engineering, Architecture and Georresources Department, Instituto Superior Técnico, Universidade de Lisboa, Av. Rovisco Pais, 1049-001 Lisbon, Portugal

**Keywords:** accelerated carbonation, alkali-activated materials, CO_2_ capture, strength development

## Abstract

This paper presents a literature review on the effects of accelerated carbonation on alkali-activated materials. It attempts to provide a greater understanding of the influence of CO_2_ curing on the chemical and physical properties of various types of alkali-activated binders used in pastes, mortars, and concrete. Several aspects related to changes in chemistry and mineralogy have been carefully identified and discussed, including depth of CO_2_ interaction, sequestration, reactions with calcium-based phases (e.g., calcium hydroxide and calcium silicate hydrates and calcium aluminosilicate hydrates), as well as other aspects related to the chemical composition of alkali-activated materials. Emphasis has also been given to physical alterations such as volumetric changes, density, porosity, and other microstructural properties caused by induced carbonation. Moreover, this paper reviews the influence of the accelerated carbonation curing method on the strength development of alkali-activated materials, which has been awarded little attention considering its potential. This curing technique was found to contribute to the strength development mainly through decalcification of the Ca phases existing in the alkali-activated precursor, leading to the formation of CaCO_3_, which leads to microstructural densification. Interestingly, this curing method seems to have much to offer in terms of mechanical performance, making it an attractive curing solution that can compensate for the loss in performance caused by less efficient alkali-activated binders replacing Portland cement. Optimising the application of such CO_2_-based curing methods for each of the potential alkali-activated binders is recommended for future studies for maximum microstructural improvement, and thus mechanical enhancement, to make some of the “low-performing binders” adequate Portland cement substitutes.

## 1. Introduction

For centuries, the construction industry’s reliance on ordinary Portland cement (OPC) as a primary construction material has been increasing with growing market demand [1]. This rising need has been principally caused by the yearly increase in the world’s population that has accelerated the demand for more housing and infrastructure [2]. In 2020, the estimated amount of OPC produced globally reached 4.3 Gt compared to 4.2 Gt produced in the previous year [3]. According to the International Energy Agency (IEA), the CO_2_ intensity of OPC production showed a 1.8% yearly increase from 2015 to 2020, accounting for around 8% of the yearly total global CO_2_ emissions [3,4]. Consequently, in the last few decades, there have been many research studies attempting to fully replace OPC with industrial by-products such as fly ash (FA) and ground granulated blast-furnace slag (GGBFS) in the production of alkali-activated materials (AAMs) in an attempt to reduce reliance on OPC and therefore reduce its environmental impacts. Despite the remarkable environmental and technological feasibility of AAMs, their chemical compositions, reactions, long-term performance, and their use as sole precursors are still widely unknown [5,6]. Amid the increasing demand for construction materials such as concrete, more aspects related to AAMs’ practical feasibility still need to be further investigated and improved [7].

The carbonation of concrete is a long-term degradation phenomenon leading to the corrosion of steel reinforcement, which is one of the main aspects that affect the durability of concrete. Therefore, several studies have been conducted to test the resistance to carbonation of concrete with and without supplementary cementitious materials (SCMs) by exposing them to an accelerated carbonation technique to obtain the results needed in a short period of time. This method has also been used to evaluate the resistance of different alkali-activated materials to carbonation to ascertain whether AAMs can replace OPC in this regard.

Nevertheless, recent studies have highlighted the performance-enhancing feature of accelerated carbonation regarding the chemical and mechanical properties of AAM as opposed to being regarded and studied solely as a degradation phenomenon. Researchers have been focusing on the benefits of accelerated carbonation as a curing technique to improve the performance of alkali-activated concrete (AAC). An induced carbonation technique has two main beneficial features on AAM: (i) fast strength development [8] and (ii) the capture/storage of CO_2_ in concrete. Besides the benefits gained in terms of performance, accelerated carbon-curing technology captures CO_2_ in concrete in stable forms of carbonates, offering a further reduction in atmospheric CO_2_. Even though some literature reviews on AAC have been published, none have focused on the effects of accelerated carbonation on the abovementioned aspects, which demonstrates the innovative character of this paper.

In this paper, previous studies related to the application of the accelerated carbonation curing method on AAM have been comprehensively reviewed. Following a defined methodology, the influence of CO_2_ on different chemical and physical properties of AAM is discussed with evidence from recent studies. Beginning with understanding the kinetics of carbonation of AAMs, the corresponding chemical reactions, and ending with the improvement in mechanical strength caused by this technique, this review paper covers crucial aspects affected by the forced carbonation method. Ultimately, the findings exposed in this paper intend to further enlighten researchers on the matter and guide future research studies by showing the importance of this curing technique on overall performance enhancement. Recently published studies on the carbonation of alkali-activated materials are compared here with earlier ones from the 20th century, highlighting the advancement in research in this field over the past two decades.

## 2. Methodology

This literature review has been prepared to better understand the influence of induced carbonation on the chemical and physical properties of alkali-activated pastes, mortars, and concrete. For this purpose, a well-determined research methodology has been followed using the Web of Science and Scopus as the main search engines. For both databases, the search was based on different combinations of the following keywords:

- Alkali-activated materials;
- Alkali-activated concrete;
- Alkali-activated mortars;
- Carbon capture;
- Carbon curing;
- Carbon sequestration;
- Carbonation curing;
- CO_2_ uptake;
- CO_2_ curing;
- Decalcification of C-(A)-S-H;
- Geopolymer.

Each search resulted in different articles related to the keywords used. A given procedure was followed to choose the studies that were further considered. To distinguish between the relevant and non-relevant studies, the titles and abstracts of studies were analysed. This analysis was based on two main criteria that each of the studies should meet:

- Articles on alkali-activated pastes, mortars, and/or concrete with alkali-activated precursors (i.e., GGBFS, FA, and other amorphous aluminosilicates binders) used as sole binders or blended with OPC or other SCMs;
- Influence of accelerated/forced/induced carbonation via gaseous CO_2_ on many properties/aspects (e.g., porosity, density, and strength) on the abovementioned materials.

Those articles that were relevant to this study were selected and collated for further analysis.

## 3. Main Factors Influencing the Mineralogical/Chemical Alterations of Carbonated Concrete

The effect of accelerated carbonation on the properties of AAM with aluminosilicate-rich precursors, such as FA and GGBFS, has been extensively studied. This method is generally implemented to test the resistance of these relatively novel materials to carbonation if used for reinforced concrete production. However, the technique of early-age-induced carbonation is being progressively investigated as a curing method with significant improvement of several properties when applied to AAC [9,10,11]. Naturally, the benefits of this technique also include reducing emissions from reforwarded CO_2_-rich flue gases and storing them permanently in materials with added value in construction.

The method involves exposing materials to an environment with an amount of carbon dioxide larger than that present in the atmosphere, thereby creating conditions wherein the AAMs are likely to undergo greater carbonation depths. Nevertheless, the chemistry behind the process is generally the same and well-documented [12] regardless of the CO_2_ content, albeit with varying carbonation rates. In conventional concrete chemistry, wherein OPC is the binder, the induced CO_2_ reacts with the Ca^2+^ ions from the pore solution or decalcified hydration products. These include the calcium hydroxide (CH) and calcium silicate hydrates (C-S-H), from the anhydrous tricalcium silicate (C_3_S) and dicalcium silicate (C_2_S) phases, to form calcium carbonate (CaCO_3_-$C\overset{-}{C}$) polymorphs [13]. The early stages of carbonation involve the decalcification of CH with subsequent formation of $C\overset{-}{C}$, the molar volume of which is greater than that of CH (36.9 cm^3^/mol vs. 33.0 cm^3^/mol, respectively). This means that slight increases in volume and in weight are expected for the carbonated region of the material. The increase in volume leads to a decrease in local porosity and, consequently, an increase in compressive strength [14,15,16]. The combination of a decreased surface porosity from CaCO_3_ precipitation in an area with previously high CH content leads to a more difficult diffusion of CO_2_, thereby slowing down the carbonation of internal regions [14]. However, in mixes with high porosity, the precipitation of calcite has little effect on the level of entry of CO_2_, leading to further decalcification of CH. Afterwards, in mixes with sufficiently spent CH, there is a greater onset of C-S-H decalcification caused by the gas [14,17,18,19,20]. Occurring in parallel to this, when the Ca^2+^ ions are consumed, negatively charged ions are created, which are eventually balanced with the formation of Si-OH groups. Later, Si-OH and Si-O-Si groups are linked together, forming a hydrated amorphous SiO_2_ [21]. The silicate chains that are formed build bridges between the neighbouring regions that are pulled together leading to the so-called carbonation shrinkage. Decalcification of aluminate ferrite monosulphate (AFm) phases also occurs during this process, wherein aluminium is released to form aluminium hydroxide or zeolites [12]. In more porous mixes, CO_2_ subsequently attacks the interior chains of C-S-H, leading to further shrinkage and eventually cracking within the microstructure. The decalcification of the C-S-H phase has been carefully studied since it is the main hydration product responsible for the strength development in conventional OPC concrete and partly for that of AAC [22].

Although knowledge of AAM carbonation is not as developed as that for OPC-based compounds, aside from a few differences presented in subsequent sections, the carbonation reactions are essentially similar. Generally, AAMs with higher Ca content have been found to present improved resistance to carbonation, higher CO_2_ uptake, and even enhanced performance [23]. Assuming equivalent microstructures with comparable porosity, each alkali-activated precursor presents different carbonation performance results depending on the chemical composition of each precursor, which may lead to different results in terms of chemical and physical properties (pores’ distribution, volume change, durability, and strength) [24]. Apart from factors related to the material itself, especially its porosity, which is governed by most of the following aspects, the main factors influencing the extent of carbonation include the nature of the precursor; the effectiveness of the alkali activator at reacting with it; the curing conditions of the materials prior to being exposed to CO_2_; as well as the specific conditions of the accelerated carbonation environment [25,26].

### 3.1. Precursors with Varying Chemical Composition

Valuable knowledge has been gathered on the morphological, mineralogical, and structural changes caused by the carbonation reaction of Ca-based phases in both conventional OPC concrete and alkali-activated materials. This information has been obtained through transmission electron microscopy (TEM) analyses [17,27], 29Si [17,28,29] and 27Al magic angle spinning nuclear magnetic resonance [30,31], mercury intrusion porosimetry (MIP) [18,26,32,33], and other infrared spectroscopies [8,34]. When SCMs normally used for alkali-activation (e.g., FA and GGBFS) partially replace OPC in conventional mixes, the kinetics of the carbonation reaction differ slightly since less CH is available to react, and there is an overlap of different reactions [20]. In such cases, CH is consumed in a pozzolanic reaction with the reactive amorphous silica and alumina from the SCM, producing C-S-H [35] and AFm phases. At the same time, for high CO_2_ concentrations, there is a fast decalcification of CH into $C\overset{-}{C}$ [36]. Additionally, the surfaces of some SCMs such as FA and GGBFS serve as thermodynamically favourable areas for the creation of nuclei of hydration products [37]. Moreover, the use of SCMs with low Ca content, which leads to an overall decrease in the Ca/Si ratio of the C-S-H and CH contents, tends to result in an even faster decalcification rate and lower formation of carbonates [17,38,39]. This is due to the proportional decrease in the carbonation-liable alkaline constituents (mostly Ca-based). This results in a decrease in the pH and a higher carbonation rate and, therefore, an increase in the carbonation depth. Naturally, the opposite may also be observed, wherein the incorporation of high-calcium FA leads to improved resistance to carbonation when compared to low-calcium FA- or silica fume-containing concrete [38,39,40,41]. Comparable results were observed for mixes containing Ca-rich basic oxygen furnace slag [42]. Additionally, the increase in Ca/Si ratio in alkali-activated materials can also lead to a decrease in the carbonation rate, not just because of the increased amount of phases that can be carbonated, but also due to the accumulation of $C\overset{-}{C}$ precipitates that can hinder further diffusion and reaction of CO_2_ in the microstructure [43,44].

Similar findings have been reported for mixes with alkali-activated GGBFS exposed to an accelerated carbonation [22]. The carbonation of the Ca-containing phases of alkali-activated GGBFS mixes can be summarized into two main stages [7,10,45]: the first is the diffusion of CO_2_ into the pores and the second involves the decalcification of the Ca-containing phases leading to the formation of $C\overset{-}{C}$. In the former stage, the phenomenon leads to a reduction in the pH due to the formation of carbonic acid-H_2_CO_3_. Here, the precipitation of sodium carbonates also occurs due to the reaction with free Na^+^ ions in the pore solution. Note that, in the second stage, some alkali-activated precursors are low in Ca and thus $C\overset{-}{C}$ formation has little significance. These precursors generally produce aluminosilicate-enriched gels with alkalis and have a poor Ca/Si ratio [46]. AAC containing these low Ca precursors entail a different “carbonation rate” (better described as pH decline), which is generally faster when compared to other precursors rich in CaO. However, the opposite phenomenon can also be observed depending on the precursor supplying SiO_2_. Behfarnia and Rostami [47] studied the effect of incorporating micro silica as a GGBFS replacement (up to 15% by weight) in the carbonation rate of AAM. This led to an overall decline in the carbonation rate. Naturally, this was a result of the much lower porosity and interconnected pores, thereby decreasing the surface area available to react within the microstructure, thus compensating for the lower Ca/Si ratio.

Bernal et al. [24] studied the influence of carbonation on the chemical composition of concrete with alkali-activated GGBFS. For this purpose, three types of GGBFS with distinct MgO contents were used to produce samples and were later exposed to different CO_2_ concentrations. The results demonstrated an inverse correlation between the slag MgO content and the carbonation depth. This result validated the role of slag MgO content in retarding the carbonation process. In the presence of reactive MgO, it hydrates to form Mg(OH)_2_, the magnesium-based counterpart of the widely known Ca(OH)_2_, which also reacts with CO_2_ to form MgCO_3_. Mg-Al hydrotalcite phases were also detected and are known to be one of the possible phases formed upon carbonation. Consequently, the carbonation performance cannot be estimated based only on the amount of CaO in the precursor, but also on other uncarbonated components containing alkaline-earth metals.

Another study [8] has investigated the influence of adding metakaolin (MK) on the CO_2_ diffusion of mortars based on alkali-activated GGBFS. The mixes prepared with slag blended with the highest amount of MK showed an increase in their susceptibility to carbonation (i.e., greater carbonation depths), whereas the mortars with GGBFS as the sole precursor presented the lowest carbonation depths. The reason for this result is that MK is generally low in CaO or MgO when compared to GGBFS. According to the authors, this led to a higher extent of formation of trona phases in the MK-free samples caused by carbonation, suggesting a greater presence of free Na^+^ ions in the pore solution. However, this was not the case for MK-blended samples, wherein the formation of aluminosilicate gel contributed to the binding of Na^+^.

Concerning the influence of the type and proportion of binder on the carbonation depth in AAC, Bernal et al. [48] compared the carbonation resistance performance of three alkali-activated GGBFS mixes containing different binder contents of GGBFS (300, 400, and 500 kg/m^3^). The results revealed that the increase in the GGBFS content increased the carbonation resistance of the specimens, while the one with the lowest GGBFS content showed the greatest carbonation depth (~70% carbonated).

In a study by Kassim et al. [49], specimens based on alkali-activated electric arc furnace slag (EAFS) showed “complete carbonation” with the use of the phenolphthalein solution test method after exposing the specimens to 28 days of accelerated carbonation. In comparison to the conventional GGBFS, EAFS proved to be considerably less reactive, resulting in specimens with significantly lower mechanical performance as well as resistance to carbonation when exposed to similar conditions [47].

Overall, the abovementioned findings suggest that, for precursors with adequate reactivity and without taking accelerated carbonation conditions or porosity of samples into consideration, the extent of carbonation mainly depends on the presence of alkali metal and alkaline-earth metal oxides and hydroxides (MO, M_2_O, MOH, and M(OH)_2_, where M usually corresponds to Na, K, Mg, and Ca, but mostly the latter, even in other phases such as C-S-H); the greater the alkaline reserve, the lower the speed of pH decline over time. The main product of this reaction is the carbonate of the abovementioned metals (i.e., MCO_3_ and M_2_CO_3_). Alkaline-earth metals (Ca and Mg) are considered to result in more stable carbonate forms, whereas the alkali metal (Na and K) carbonates may be soluble when exposed to water. Nevertheless, all are capable of further refining the microstructure, thereby leading to densification in cases wherein there was not an extensive decalcification (assuming Ca as the predominant element) of existing phases.

### 3.2. Alkali Activator

Alkaline activators obviously play a vital role in the production of AAC. They are used to trigger the reactions between the reactive silica and alumina in the precursor and the activating solution, which bind and harden the concrete mix. The reaction in alkali-activated materials is typically broken down into four stages according to Davidovits’ model and as described by Duxson et al. [50,51]. These stages include dissolution, condensation, polycondensation, and crystallization of the gels. The initial stage of the reaction involves the breaking down of aluminosilicate materials into reactive silicate and aluminate monomers represented as [Si(OH)_4_]^−^ and [Al(OH)_4_]^−^, respectively. The bonds between Si-O-Si and Al-O-Al in the aluminosilicates are dissolved by a strongly alkaline solution with a pH of around 14, leading to the formation of a colloidal phase. In simpler terms, the alkaline solution weakens the bonds in the aluminosilicates, creating several dispersed insoluble particles in suspension in the mix solution [50,51,52]. The creation of a colloidal phase leads to a water elimination process through a nucleophilic substitution reaction. The [Si(OH)_4_]^−^ and [Al(OH)_4_]^−^ monomers, which have a charge of −1, interconnect through the attraction between the OH groups of silicate and the Al ions of the aluminate. This initiates the chemical equilibrium known as condensation, resulting in intermediate compounds. This stage also results in the formation of an unstable aluminosilicate species accompanied by the release of water molecules [51,53]. The process continues with further water release and the formation of initial gels. The pursuit of balance continues but cannot be achieved due to both aluminates and silicates possessing negative charges. The presence of positively charged alkali metal ions, such as Na^+^ or K^+^, in the alkaline solution becomes crucial in this stage. These ions provide balance to the negative charges of the unstable gels formed, causing a reorganization in the structure of the intermediate compounds and leading to the creation of a more durable final product [54]. Polycondensation of the gels then takes place, which may result in crystallization and the formation of stable gels in the final geopolymer structure. Some authors refer to amorphous gels as N-A-S-H (hydrated sodium aluminosilicate) and crystalline or semi-crystalline phases as zeolites [55]. During this stage, the material begins to harden, gaining mechanical strength and other desired properties of alkali-activated materials [51,52,54].

The extent of carbonation depends on the CO_2_ concentration and its diffusion through the specimen. The rate at which these two features affect the overall carbonation of the material is contingent on the porosity of the material, which in turn is largely dependent on the type and concentration of alkali activator used. Shi et al. [17,27] reported the microstructural changes in alkali-activated GGBFS mortars activated by NaOH and a sodium silicate solution with different Na_2_O/precursor dosages and SiO_2_/Na_2_O ratios steam-cured for 48 h and subsequently carbonated for 56 days at 3 ± 0.2% CO_2_ at atmospheric pressure. The results showed that the increase in the activator’s Na_2_O content and SiO_2_/Na_2_O ratio led to increased resistance to carbonation, the magnitude of which was greater with the increase in the latter. This was the result of a reduction of the hardened material’s total porosity and average pore size.

Li et al. [22] also analysed the effect of using different dosages of the alkaline activator on the structural changes in carbonated alkali-activated GGBFS mortars. The materials activated with NaOH alone showed greater resistance to carbonation (measured via residual strength) than those with NaOH and a sodium silicate solution. Increasing the SiO_2_/Na_2_O ratio of the alkaline activator resulted in the formation of C-S-H with lower Ca/Si. This led to an increased decalcification rate of C-S-H, as there was a small amount of CH present. The authors of another study reached a similar conclusion [56].

Moreover, Carvalho et al. [57] assessed the effect of optimising the alkaline solution on the mechanical performance of alkali-activated mortars made with municipal solid waste incinerated bottom ash (MIBA) as a precursor and cured under forced carbonation. The study revealed the effect of using different NaOH and Na_2_SiO_3_ proportions on flexural and compressive strengths. The carbonated mixes activated with NaOH alone showed higher flexural and compressive strengths compared to carbonated mixes activated with both NaOH and Na_2_SiO_3_. In addition, the increase in NaOH content led to increases in mechanical performance. Moreover, Casanova et al. [58] reported an increase in compressive strength with carbonated MIBA mortars activated with NaOH only, thus reporting a decrease in the carbonation resistance.

Furthermore, Avila [59] studied the effect of different NaOH and Na_2_SiO_3_ proportions on the mechanical performance of alkali-activated MIBA and FA mortar specimens cured under forced carbonation. The results showed how varying the Na_2_O/binder and SiO_2_/Na_2_O ratios can largely influence the porosity profile of the hardened specimen, affecting its carbonation resistance. This conclusion was confirmed after reporting different compressive strengths of carbonated FA and MIBA specimens when different alkaline solution ingredients proportions were applied.

Kassim et al. [49] investigated the influence of using different combinations of NaOH and Na_2_SiO_3_ for the alkaline solution to improve the mechanical and microstructural performance of EAFS mortars cured under forced carbonation. Similar to other studies, the increase in Na_2_O/binder and SiO_2_/Na_2_O ratios created a denser microstructure with reduced porosity, increased the mechanical performance, and enhanced the carbonation resistance of the specimen.

The influence of the alkaline activator on the carbonation of AAC is a complex issue that must be evaluated from different perspectives. The use of optimum alkali activator contents, chosen based on maximized performance, correlates with decreased porosity, thereby hindering CO_2_ diffusion and reaction due to the lower surface area available to facilitate reactions. For this reason, AAM with higher SiO_2_/Na_2_O ratios, which usually present better performance, have presented higher resistance to carbonation. Nevertheless, from the perspective of increasing CO_2_ uptake, the optimum values of the alkaline activator’s Na_2_O content and SiO_2_/Na_2_O ratio shift when comparing the top performance of uncarbonated specimens with carbonated ones [49].

### 3.3. Curing Conditions

The conditions of curing prior to the CO_2_ exposure period play a crucial role in influencing the CO_2_ diffusion mechanisms in AAC. Different alkali-activated binders are distinct in their chemical compositions and reactions, and different curing conditions and periods lead to different microstructural profiles. Table 1 presents a compilation of results from several studies regarding the carbonation depth of alkali-activated specimens measured using the phenolphthalein pH indicator method. Nedeljkovićet al. [45] evaluated the CO_2_ penetration of AAC samples prepared with FA and GGBFS as precursors when exposed to different curing conditions. The curing regime followed in this study was composed of three steps: wet curing, dry curing, and CO_2_ curing. During the wet curing, some of the specimens were sealed while the rest were not; however, during the dry and CO_2_ curing periods, all specimens were unsealed. Naturally, the results showed that the diffusion of CO_2_ was faster in unsealed samples when compared to the sealed ones. The most probable reason for this result is the microstructure modification of the unsealed samples caused by the dissolution and migration of Na^+^ ions. This change in the microstructure, which likely included greater interconnected porosity, facilitated the diffusion of CO_2_ through the unsealed samples, leading to increased carbonation depth. Similar results were reported by other authors [9,60]. Furthermore, regardless of the curing condition, GGBFS-containing specimens presented a much lower carbonation depth, and thus an improved resistance to carbonation, when compared to FA specimens. This is most likely due to the greater presence of CaO in GGBFS, which can act as a buffering agent for the pH of the pore solution, thereby increasing resistance to carbonation.

In a study by Behfarnia and Rostami [47], similar observations were made in terms of the effect of a water-based vs. a plastic film-covered curing before having subjected the specimens to accelerated carbonation. The alkali-activated specimens, which comprised a blend of GGBFS and micro silica at 85–95% and 15–5%, respectively, were placed in a carbonation chamber with a ~4% CO_2_ content for 28 days. Before this step, the specimens had been cured either directly in water or wrapped in plastic film. The specimens cured using the latter technique presented higher carbonation depths that were inversely proportional to the specimens’ compressive strength, meaning that water-based curing is apparently more effective than the plastic-cover curing.

Morone et al. [61] studied the performance of AAM based on basic oxygen furnace (BOF) slag. Different samples of BOF slag were prepared using different types and concentrations of alkali activators. These samples were subjected to distinct curing conditions (temperatures of 20 °C, 22 °C, and 50 °C; curing durations of 3, 7, and 28 days; relative humidities of 75% and 98%; and 5% CO_2_ concentration). It was interesting that all the samples carbonated and cured at 50 °C showed a constant carbonation trend over time regardless of the varying conditions. The reason for this carbonation “blockage” is the early formation of $C\overset{-}{C}$ at the outer layer of the samples that blocked any further diffusion of CO_2_. Moreover, the maximum amount of CO_2_ captured was equal to 5.2 wt% reported for samples exposed to accelerated carbonation at 20 °C after interpreting the thermogravimetric analysis (TGA) and the differential thermal gravimetry (DTG) results to identify the reaction products. The authors also observed that the increase in temperature of the reaction led to increased dissolution of Ca while reducing the solubility of CO_2_ [62]. Other studies confirmed the reduction in the CO_2_ solubility, but only when exposing the specimens to accelerated carbonation at temperatures exceeding 60 °C, at an atmospheric pressure [61,63].

The carbonation process of AAC significantly depends on its microstructure. Applying proper curing conditions is likely to lead to a denser and more uniform microstructure capable of slowing down carbonation. The most appropriate conditions for this to occur are those that prevent excessive humidity exchanges with the surrounding environment. Sealed curing or being placed in an environment with relatively high humidity (though not saturated) are likely ideal. AAM placed in a relatively dry environment leads to the loss of a notable amount of water that could otherwise have been part of the C-(N-)A-S-H gels. The worst environment was found to be that in which the specimens were in direct contact with water; this causes the dissolution of the highly soluble and poorly linked Na in the microstructure, thereby causing leaching and degradation.

**Table 1:** Carbonation depth measured via pH indicator of alkali-activated materials exposed to accelerated carbonation under different conditions.

Main Precursor (Secondary)	Precursor Content (% Total Binder)	Conditions of Accelerated Carbonation				Carbonation Depth (mm)	Refs.
		Temperature (°C)	Relative Humidity (%)	CO_2_ Concentration (%)	Exposure Time		
FA+GGBFS	0–100	20	60	1	500 days	0–20 ^a^	[45]
GGBFS	100	30 ± 1	70 ± 3	7 ± 0.5	16 days	17–34 ^b^	[64]
GGBFS	100	-	43.2	100%	4 months	1–10 ^c^	[10]
GGBFS	100	25 ± 2	65 ± 5	1 ± 0.2	1000 h	~21–48 ^d^	[48]
GGBFS	100	23 ± 2	65 ± 5	7	16 days	16–38	[65]
GGBFS	100	-	70	20	4 months	13	[66]
GGBFS (micro silica)	85–95	22 ± 3	65 ± 5	4 ± 0.1	28 days	4.8–6.7 ^e,^*	[47]
						8.7–14 ^f,^*	
GGBFS (MK)	80–100	20 ± 2	65 ± 5	3 ± 0.2	340 h	15–19 ^g^	[8]
	80–100	25 ± 2	(50–80) ± 5	1 ± 0.2	500 h	5–10 ^h^	[67]
EAFS	100	23 ± 2	65 ± 5	5	28 days	20 ^i^	[49]

Note: ^a^ FA mixes with significantly greater depth vs. GGBFS mixes (previously with (un)sealed curing-sealed led to lower depths; complete carbonation in most specimens after a short period of time); ^b^ 340–512 kg/m^3^ of precursor (inversely proportional to carbonation depth); ^c^ NaOH or sodium silicate as activators (latter led to greater carbonation); ^d^ 300–500 kg/m^3^ of precursor (inversely proportional to carbonation depth); ^e^ water-cured before carbonation; ^f^ plastic film-covered before carbonation; * increased micro silica content led to lower depths; ^g^ SiO_2_/Na_2_O ratio varied between 2.4 and 1.6-lower ratios led to greater carbonation (increasing the MK content led to complete carbonation); ^h^ 65% RH led to maximum depths (increasing the MK content further increased carbonation); ^i^ completely carbonated specimens.

### 3.4. Conditions of Accelerated Carbonation

Apart from the aforementioned aspects, the actual conditions, wherein the carbonation takes place, obviously also have a considerable impact on the reaction rate. The most important variables are CO_2_ concentration, the pressure of the surrounding environment, relative humidity (RH), and temperature. Hence, finding the optimum exposure conditions and period for each precursor are key to ascertaining the carbonation rate.

Discarding the effect of the materials’ properties, the concentration of CO_2_ is the most critical variable influencing the carbonation front of cementitious materials (Table 1). Some studies have shown that CO_2_ concentration affects the chemical composition and microstructure of AAC by comparing the results to those of similar specimens carbonated using a lower CO_2_ concentration [7,67]. Others [68] stated that the influence of CO_2_ concentration is even higher when the percentage of other types of precursors (generally low on CaO) is high. Bernal et al. [7,67] carried out a comprehensive analysis of the effect of different CO_2_ concentrations on the mineralogical changes in AAM. At higher concentrations, because of an accelerated carbonation stage, the aluminosilicate gel is highly polymerised. This generally happens in systems with high CaO content such as GGBFS. On the other hand, for (class F) FA-based mixes, the decalcification–degradation mechanism of the pre-existing structure has little significance, and the carbonation reaction occurs with alkali salts present in the pore solution (usually sodium carbonates in Na-based activators). Thermogravimetry and NMR analyses have shown little change in N-A-S-H phases after accelerated carbonation regardless of the CO_2_ content. The C-A-S-H phase, on the other hand, decalcifies similarly to other Ca-based phases.

Regarding the effect of RH during the accelerated CO_2_ curing stage, all identified studies used an RH of 50–70% (Table 1) as this range is known to be optimum for a more accelerated carbonation rate [5,69]. However, the carbonation of AAC at a low RH can result in drying shrinkage that leads to the formation of micro-cracks, which subsequently lead to a faster reaction rate of CO_2_ [67,70]. Other research studies (Table 1) have also confirmed that the best CO_2_ binding rate happens at RH between 50 and 70% with a temperature range of 20–25 °C [26,71].

In conventional OPC-based materials, the carbonation rate usually increases with the increasing pressure of the surrounding CO_2_-enriched environment. Hyvert et al. [72] studied the influence of CO_2_ pressure on the carbonation depth and the dependency of the C-S-H carbonation rate on the carbon pressure applied. Four different rates of CO_2_ partial pressures were used (~0.03%-atmospheric, 10%, 25%, and 50% of P_atm_) and applied for mortar samples cast as cylinders with 11 cm diameters and 22 cm heights made with slag as SCM. The results showed that the CO_2_ pressure applied not only affects the depth of carbonation, but also the chemical performance of the mortars. The mercury intrusion analysis indicated a decrease in the porosity in the carbonated zone when the CO_2_ pressure was increased. In addition, the TGA/DTA analysis showed that this decrease in porosity was linked to the high rate of carbonation under high pressures compared to a lower carbonation rate at lower pressures. Moreover, from the X-ray diffraction (XRD) and TGA/DTA results, the study concluded that the C-S-H carbonation rate depends on the CO_2_ pressure applied. However, these findings cannot be directly extrapolated to AAM. Jun et al. [73] exposed alkali-activated GGBFS pastes to different carbonation conditions including carbonation at 20% CO_2_, 70% RH, and 25 °C and 3-bar 99% pure CO_2_ at 25 °C and 85% RH. All mixes were activated with NaOH and sodium silicate solution. Counterintuitively, the amount of $C\overset{-}{C}$ in the 20% CO_2_ environment decreased with increasing exposure (8.37%, 6.96%, and 2.27% after 4 h, 24 h, and 7 days of exposure, respectively), as confirmed via thermogravimetric analysis. In parallel, the authors also witnessed an increase in C-S-H over time. The specimens exposed to CO_2_ under pressure, despite presenting increasing amounts of $C\overset{-}{C}$ over time, were lower than those of the aforementioned specimens (i.e., 1.43–4.07%).

The conditions of accelerated carbonation are key factors affecting the carbonation reaction. Variable factors such as CO_2_ concentration, pressure, relative humidity, and temperature can significantly affect the microstructure of AAC. The relative humidity has usually been maintained at ~60% as this will likely lead to greater carbonation depths similar to that of OPC concrete. Increased CO_2_ concentration and pressure are usually directly proportional to greater carbonation depths within a given timeframe. However, some findings showed otherwise; the abundance of CO_2_ may cause the formation of excessive amounts of carbonic acid that hinders $C\overset{-}{C}$ formation and may even dissolve those that were already precipitated because of the accelerated carbonation process. This might be circumvented with strict control over the relative humidity; decreasing it to a more appropriate level leads to the oversaturation of Ca^2+^ within the acidic solution, thus leading to the spontaneous precipitation of $C\overset{-}{C}$.

## 4. Strength Development of AAM Subjected to Accelerated Carbonation

Most of the studies concerning the carbonation of AAC have indicated changes in the microstructure, the extent of which varies depending on the aforementioned factors. Naturally, these microstructural changes affect materials’ mechanical performance. Despite the notable amount of literature on the carbonation of AAM, the strength behaviour has not been the focus of many studies. It is well known that exposing OPC-based materials to CO_2_ leads to an overall strength increase. However, the micro- and thus macrostructural mechanics of AAM may differ significantly, wherein both strength declines and enhancements have been reported. The former case often applies [8,9,10,33,65,66,67], though many have pointed out that this may actually be beneficial from a mechanical perspective [49,57,58,59,73,74] aside from the capture and storage of CO_2_ (Table 2).

Nedeljkovic [9] studied the resistance to carbonation of alkali-activated GGBFS under natural and accelerated conditions. The authors identified one major carbonation reaction, which was the precipitation of $C\overset{-}{C}$ polymorphs from the decalcification of C-(N-)A-S-H phases. Almost all specimens presented a decline in compressive strength after being subjected to the accelerated carbonation regime. However, the decrease was relatively low (~5% in mixes with compressive strength values between 100 MPa and 120 MPa) as there was a blockage of CO_2_ diffusion after the first 28 days of accelerated carbonation, making the samples highly resistant to carbonation, regardless of the exposure conditions.

In a study by Shi et al. [33], wherein the influence of Na_2_O/precursor and SiO_2_/Na_2_O ratios on the carbonation resistance of GGBFS mortars was analysed, contrasting findings were reported. On the one hand, increasing both ratios led to significantly high compressive strength before carbonation (over 100 MPa); however, with progressive exposure to a ~3% CO_2_ environment, compressive strength dropped significantly (~30% for mixes with Na_2_O/precursor and SiO_2_/Na_2_O ratios of 8% and 2.0, respectively). On the other hand, even though the use of NaOH alone to activate GGBFS led to lower initial compressive strength, after the accelerated carbonation, the compressive strength increased ~35% from around 45 MPa for mixes with a Na_2_O/precursor ratio of 8%. The degradation in the former mixes can be explained by the carbonation of C-(A-)S-H phases, which led to a reduction in the molar volume, thereby increasing the total porosity and average pore size, thus resulting in a reduction in performance. For the mixes with NaOH alone, the increased compressive strength was attributed to the carbonation of katoite-C_3_AH_6_-, which resulted in increased molar volume, and thus a reduction in total porosity and average pore size. Bernal et al. [8,65] also reported similar findings for alkali-activated GGBFS mixes wherein the loss of performance was a result of the decalcification of C-(A-)S-H phases that caused a reduction in compressive strength. Puertas et al. [10] also studied the effect of different activators on the carbonation of activated GGBFS and reported the same trend as that observed by Shi et al. [33]. A greater loss in cohesion was observed for mixes activated with NaOH and sodium silicate solution from the decalcification of C-S-H phases in comparison to those produced with NaOH alone. In the latter, greater precipitation of $C\overset{-}{C}$ had been observed, resulting in a decrease in total porosity and average pore size, consequently leading to enhanced performance.

Li et al. [22] also studied the carbonation of alkali-activated GGBFS mortars made with alkaline activators with different contents and presented findings somewhat similar to those of Shi et al. [33]. Increased resistance to carbonation was observed for mixes made with NaOH alone wherein a lower loss in performance was observed in comparison to mixes with NaOH and sodium silicate. In the latter, as stated in Section 3.2, the carbonation rate of C-S-H with a lower Ca/Si ratio was much more intense. The authors found that $C\overset{-}{C}$ particles precipitated mainly in the micropores, including gel pores and spaces in the C-S-H interlayer and that there was a limited effect on the macropores, causing shrinkage. Additionally, as the crystallization of $C\overset{-}{C}$ was restricted to the smaller-sized pores, there was a decline in compressive strength.

Bernal et al. [67] also reported a decrease in the compressive strength of mixes made with alkali-activated GGBFS and GGBFS + MK after exposing them to accelerated carbonation (1 ± 0.2% CO_2_ and 25 °C) for 250 or 500 h. Before carbonation, the compressive strength of the 100% GGBFS sample was 63 MPa; however, after 250 h of accelerated carbonation at 50% RH, the residual compressive strength dropped to 54 MPa (15% decrease). The strength loss was even higher for mixes with MK; after 500 h, the strength loss was around 40% when cured at 50% RH.

Contrary to previous findings, Han and Kim [74] reported a consistent increase in compressive strength in alkali-activated GGBFS pastes after exposing them to a 20% CO_2_-containing environment for 3 days. This was observed in mixes with both sodium silicate solution and/or NaOH alone as the activators. Although no thermal curing stage had been applied, the authors observed that simply drying the specimens for a short period of time (3 h in a vacuum chamber) was enough to improve CO_2_ diffusion and thus achieve strength enhancement; however, a decline was observed when the drying time was extended for too long (24 h in vacuum). The authors also reported that significantly enhanced compressive strength of specimens (with no exposure to CO_2_) was observed in mix designs including a notable portion of sodium silicate as the activator. The C-S-H phase formed in these activated GGBFS is likely to show a higher level of cross-linking than that formed in mixes activated with NaOH and/or Na_2_CO_3_ [75].

Jun et al. [73] studied the effect of curing alkali-activated GGBFS pastes under pressure (i.e., 3-bar and 99% pure CO_2_) and in atmospheric pressure with 20% CO_2_. As stated in Section 3.4, despite having been exposed to a significant amount of CO_2_ under pressure, the amount of $C\overset{-}{C}$ was not significant, and the mechanical performance was even lower than that of specimens cured at 25 °C and 85% RH. However, those cured at 20% CO_2_ presented an increase of ~12% in compressive strength after 45 days when compared to specimens without carbonation.

Carvalho et al. [57] reported a considerable strength increase after exposing alkali-activated MIBA mortar specimens to accelerated carbonation (at 23 ± 3 °C, 60 ± 5% RH, and 5.0 ± 0.1% CO_2_) for a period ranging between 28 and 91 days. According to this study, all MIBA mixes, each prepared with an alkaline solution having different NaOH and sodium silicate solution proportions, showed an increase in flexural and compressive strengths. The highest increase in mechanical strength was seen for two mixes that both had the highest Na_2_O/precursor ratio (24.2%), one with a SiO_2_/Na_2_O ratio of 1.0 and the other without a Na_2_SiO_3_ solution. The mix with no sodium silicate content in the solution showed an increase in compressive strength of ~166% and ~63% in flexural strength after 91 days of CO_2_ curing. Moreover, the mix with a Na_2_SiO_3_ solution in its content showed an increase of ~125% in compressive strength and ~120% in flexural strength after 91 days of CO_2_ curing. The study reported an increase in mechanical strength from 28 to 56 days of CO_2_ curing; however, a loss in strength started to be seen after day 56. One possible reason for this initial increase and subsequent decrease in strength is that $C\overset{-}{C}$ and amorphous SiO_2_ gel are formed during the carbonation curing process, bonding together to create a denser matrix that is responsible for the strength increase in the specimen [76]. However, the loss in strength after long exposure to CO_2_ is linked to the tensions generated around the unreacted particles caused by the carbonation process, creating micro-cracks due to shrinkage [7].

Casanova et al. [58] studied the strength development of alkali-activated MIBA after exposure to a predefined curing regime that included a carbonation curing stage at 23 ± 3 °C, 60 ± 5% RH, and 5.0 ± 0.1% CO_2_ for 28 days and 91 days. Concerning the FA control specimens, a clear strength increase of 20 MPa was reported when exposed to 28 days of accelerated carbonation compared to the uncarbonated ones. All FA specimens cured with CO_2_ for 91 days showed an increase in strength of 18–36% when compared to the same uncarbonated specimens. Regarding the MIBA specimens, a significant increase in strength of 275–306% was reported, with 28 MPa as the highest compressive strength recorded after 91 days of carbonation.

Avila [59] confirmed the strength development of alkali-activated MIBA and FA after exposure to accelerated carbonation at 5% CO_2_, 20 °C, and 60% RH for 7, 14, and 28 days. The most significant gain in compressive and flexural strengths was seen for carbonated mixes (28 days exposure) with 100% MIBA, i.e., a 140% increase in compressive strength (8.08 MPa to 19.1 MPa) compared to uncarbonated ones. For the same mixes, a ~185% increase in flexural strength was reported as well after exposure to 28 days of carbonation (1.58 MPa to 4.50 MPa) compared to the same uncarbonated mixes. Regarding the mixes made with 100% FA, an increase of 30% in compressive strength was reported after 28 days of accelerated carbonation.

Kassim et al. [49] conducted an experimental study on alkali-activated EAFS mortars exposed to accelerated carbonation for 28 days (5 ± 0.2% CO_2_, 23 ± 3 °C and 60 ± 5% RH). A significant increase in compressive strength of ~800% (from 3.9 MPa to 31 MPa) and a ~500% increase in flexural strength (from 1.6 MPa to 7.85 MPa) was reported for specimens exposed for 28 days to accelerated carbonation.

## 5. Carbon Dioxide Sequestration Potential

Apart from the importance of CO_2_ curing in potentially improving the properties of AAC, this technique may have considerable environmental benefits. The carbonation reaction that occurs between the CO_2_ and the reactive compounds fixes the molecule in stable carbonates such as calcium and magnesium carbonates [38,77]. Thus, the rapid carbonation of AAM can be considered a viable method to further enhance the performance of such materials, especially those containing precursors that are likely to present decreased performance, as well as to capture and store CO_2_.

Some research studies [7,9,21,22,61] have been conducted to understand the influence of the type of binder, exposure condition, and exposure time on the CO_2_ uptake capacity of AAC. Nedeljkovic et al. [9] investigated the carbonation mechanism of alkali-activated GGBFS pastes exposed to accelerated carbonation. The CO_2_ uptakes of the samples were studied using FTIR spectroscopy and TGA. After being exposed to 28 days of carbonation, the CO_2_ uptake reached 14 wt% of the precursor.

Morone et al. [61] investigated the possibility of capturing CO_2_ in alkali-activated BOF steel slag pastes through exposure to accelerated carbonation with 5% CO_2_ for durations of 3, 7, and 28 days. The mineralogical and thermal analysis of the TGA and DTG results showed the formation of a C–S–H-like phase. The results indicated a maximum CO_2_ uptake of 6% according to weight of the precursor in the formation of $C\overset{-}{C}$ in the BOF samples activated with a solution with NaOH and Na_2_CO_3_. This amount was 5% according to weight for samples activated with NaOH and sodium silicate solution.

Furthermore, Bernal et al. [7] investigated the effects of subjecting alkali-activated FA and BFS pastes to distinct CO_2_ concentrations on their chemical and microstructural profiles. The results were analysed to understand the formation of different carbonation products as well as the possibility of these binders being used as a carbon sink. The reduction in the mass loss intensity reported using the TGA technique was related to the dehydration of the binding gel and decarbonation of CO_2_-containing products such as $C\overset{-}{C}$. Although the “fixed” CO_2_ content was not quantified in the study, the analysis proved the ability of FA+BFS systems to capture CO_2_ through carbonation products such as $C\overset{-}{C}$.

Another study [78] indicated a carbon sequestration efficiency of 22.6% after exposing alkali-activated FA pastes to accelerated carbonation at a pressure of 4 bar with 100% CO_2_ for a period ranging between 3 h and 3 days. The chemical and physical characteristics of the FA pastes were analysed by applying the TGA technique. The results indicated that, after 3 days of CO_2_ curing, the highest CO_2_ capture was 4.8 wt% of the precursor. The results also showed that, when the total CO_2_ curing time was increased to 3 days, ~80% of carbonated products appearing as efflorescence before were stored in the matrix.

Cai [21] also performed a TGA of alkali-activated GGBFS concrete. Loss in mass was observed at relatively low temperatures (starting at ~100 °C) due to the loss of water in the interlayer of C–(A)–S–H as well as its dehydration, and decomposition of $C\overset{-}{C}$ formed during the CO_2_ curing period. These results indicated the presence of a notable amount of captured CO_2_ in the sample. Other studies reported similar findings [73,74].

Moreover, the DTG curves of Li. et al. [22] indicated several endothermic peaks corresponding to the decalcification of C–S–H in the mix and the thermal decomposition of $C\overset{-}{C}$ in alkali-activated GGBFS mortar samples. DTG showed mass losses ranging between 3.17% and 4.64% in slag samples, indicating the presence of a considerable amount of $C\overset{-}{C}$ in the matrix. Similar results were reported by other studies on alkali-activated slag, FA, and MIBA [33,79,80].

## 6. Conclusions

Restricting the diffusion of carbon dioxide in conventional concrete has been one of the main directives to further extend the durability of steel-reinforced materials. However, given the significance of the gas towards the greenhouse effect and, consequently, the recently adopted carbon taxation system to mitigate its emission, an additional paradigm shift is required concerning the stigma associated with the carbonation of concrete, especially since the latter is one of the major world emitters.

This paper has the primary purpose of providing a greater understanding of the carbonation of alkali-activated materials via gaseous CO_2_ by gathering and analysing the most relevant research documents on the matter. The effects of several aspects on the carbonation of the materials were analysed, including precursors with different chemical compositions, the varying concentrations of the alkaline activator, the curing regimen applied before the carbonation, as well as the actual conditions of the CO_2_-rich environment. Generally, the carbonation of alkali-activated materials is mostly relevant when a calcium-rich precursor is present as the chemical process occurs via the decalcification of Ca-bearing phases. Nevertheless, notable micro-and macro-structural changes may also occur in low-Ca-containing precursors. In the former case, the main result of the reaction is the precipitation of calcium carbonate polymorphs in empty spaces adjacent to the previously calcium-containing phase, sometimes contributing to localized densification. The remaining SiO_2_ from the decalcified phases may form amorphous SiO_2_ gels, which bind together with nearby equivalent formations, thereby creating a more interconnected and denser matrix. In the latter case, which corresponds to low Ca/Si systems, calcium carbonate precipitation plays a small role in the process, and evidence suggests that most products of the carbonation are a result of the reaction with free sodium ions (or potassium, depending on the alkaline earth metal in the activator) in the pore solution. This translates into the precipitation of sodium carbonates in previously empty spaces, thereby also contributing to significant local densification.

The phenomenon of carbonation in alkali-activated materials is often directly compared with that in conventional concrete. However, the carbonation rate measured via the phenolphthalein pH indicator test method often offers misleading results concerning the actual effect of CO_2_ and does not allow accurate prediction of the potential CO_2_ uptake. Indeed, this process occurs in parallel with the activation of the precursor itself wherein there is an ongoing consumption of hydroxide ions from the alkaline activator towards the formation of calcium (sodium) aluminosilicate hydrates. Both contribute significantly to the decline in the pore solution’s pH. The literature includes specific examples demonstrating this wherein specimens were tested using the pH indicator method; the carbonation depth of specimens with notably lower resistance and higher porosity was practically inexistent (a pinkish hue was seen on most of the broken surface sprayed with the pH indicator) in comparison with other specimens with significantly better performance but that present apparently entirely carbonated profiles. In the former, a significantly greater amount of hydroxide ions remains unreacted in the matrix’s pore solution due to the presence of a poorly reactive precursor, thereby increasing the material’s pH level. This shows how the processes work simultaneously, often with an equivalent degree of importance towards the decline in the material’s pH. Furthermore, specimens exposed to atmospheric CO_2_ after relatively low exposure periods are also likely to present “high carbonation” when using the aforementioned method. Therefore, the use of a pH indicator is not an entirely adequate approach for the analysis of the carbonation depth of alkali-activated materials and must be complemented with other methods such as infrared spectroscopy and thermogravimetric analysis.

As mentioned above, there are significant changes in the microstructure due to carbonation, which also affects the mechanical performance of alkali-activated materials. Early studies on the carbonation of these materials, mostly comprising ground granulated blast furnace slag, showed a notable decline in performance, thereby discouraging further research on the effect of the gas. This decline is caused by the generalized decalcification of the C-A-S-H phases. Even though calcium carbonate is a major outcome of the reaction, it leads to a lower molar volume in comparison to the original calcium-bearing phases and thus results in greater porosity and worse mechanical behaviour. However, more recent research suggests that this tends to occur in low Ca/Si systems prompted by the use of SiO_2_-rich alkaline activators. Those using sodium (potassium) hydroxide only are likely to exhibit notable strength increases for systems with either high or low amounts of calcium. Additionally, strength enhancement resulting from the forced carbonation process is more likely to occur in specimens with medium to low strengths. In such materials, the interconnected porosity and surface area are deemed higher, which not only allows for more generalized carbonation but also for the formation of available space for the precipitation of calcium carbonates, avoiding the occurrence of internal micro-cracks that affect mechanical performance. Therefore, this provides a unique opportunity to valorise some industrial by-products (e.g., electric arc furnace steel slag, municipal solid waste incinerator bottom ash, and waste glass rejects), which would be otherwise considered waste, in the production of alkali-activated materials that additionally can be considered a carbon sink.

As a recommendation, aspects such as alkaline solution type and concentrations and curing and accelerated carbonation conditions should be optimised for each potential alkali-activated material, taking into consideration their different chemical properties, and thus benefiting from maximised microstructural and mechanical property enhancements.

## 7. Future Work

The accelerated carbonation curing of alkali-activated materials appears to be an attention-grabbing method capable of boosting the mechanical performance of alkali-activated concrete and shifting their use from basic low-performance applications to more performance-demanding ones. However, attention should be given to the following recommendations for future research on the strength development of accelerated carbonation of alkali-activated concrete:

- Further research: Continued in-depth exploration is required for an extended examination of the effect of accelerated carbonation curing techniques on the strength development of alkali-activated materials. Additional studies should investigate influencing factors such as the composition of the binder, curing conditions, and the microstructure of concrete;
- Consistent protocols: Standardized protocols for the accelerated carbonation process of alkali-activated concrete should be developed and adopted, guaranteeing consistent and reliable results. This could include the development of more advanced equipment and the optimization of the curing conditions such as the pre-curing environments, carbon dioxide concentration, temperature, surrounding pressure, and relative humidity;
- Comparative studies: Comparative studies should be carried out to evaluate the influence of induced carbonation on the micro- and macro-structure of alkali-activated concretes produced using different types of precursors and cured under different conditions. Such a step will help identify the most effective curing conditions for each type of binder for a given alkaline solution constituent’s type and proportions;
- Materials application: Future investigations may explore the potential applications of alkali-activated materials cured under forced carbonation. Future studies are recommended to assess the suitability of alkali-activated materials in diverse structural and non-structural applications such as precast concrete elements. Additionally, we recommend conducting a life-cycle assessment to study the economic and environmental feasibility of using alkali-activated concrete cured under induced carbonation by creating a comparison with conventional materials such as ordinary Portland cement concrete. Such a step will help identify alkali-activated materials’ potential areas of application, leading to wider industrial acceptance and usage.

To conclude, the accelerated carbonation curing method is a technique that seems to be bringing the alkali-activated concrete closer to being used in structural applications by enhancing many crucial properties. Supplemental investigation and standardization of the method and its conditions are needed for more consistent and reliable results.

## Figures and Tables

**Table 2:** Variation in compressive and flexural strengths of AAMs subjected to accelerated carbonation.

Precursor	Compressive Strength			Flexural Strength			Ref.
	Before Carbonation (MPa)	After Carbonation (MPa)	Variation in Strength (%)	Before Carbonation (MPa)	After Carbonation (MPa)	Variation in Strength (%)	
GGBFS	45.0	61.0	35	-	-	-	[33]
GGBFS	21.0	30.0	43	-	-	-	[74]
GGBFS	24.8	122.0	393	-	-	-	[73]
GGBFS	63.0	30.0	−52	-	-	-	[8]
GGBFS	110.0	105.0	−5	-	-	-	[9]
GGBFS	80.0	69.0	−14	-	-	-	[10]
GGBFS	90.0	63.0	−30	-	-	-	[33]
GGBFS	53.0	48.0	−10	-	-	-	[66]
EAFS	3.9	31.0	800	1.6	7.9	500	[49]
MIBA	6.4	17.0	166	3.0	4.8	63	[57]
MIBA	9.0	27.0	200	-	-	-	[58]
MIBA	8.1	19.1	140	1.6	4.5	185	[59]
FA	33.0	43.0	30	8.0	13.0	63	[59]
FA	50.0	68.0	36	-	-	-	[58]

Note: values in MPa were rounded to the nearest decimal place, and values representing percentages were rounded to the nearest whole number. Positive variation in strength represents strength increase, while a negative percentage variation indicates a decrease.

## Data Availability

The data presented in this study are available on request from the corresponding author.
